# Association of smoke exposure with cognitive function trajectories among middle and old-aged adults: evidence from the China Health and Retirement Longitudinal Study

**DOI:** 10.7189/jogh.15.04150

**Published:** 2025-05-05

**Authors:** Rulin Li, Lanjun Luo, Changwan Yuan, Qi Zhu

**Affiliations:** 1 Key Laboratory of Digital-Intelligent Disease Surveillance and Health Governance, North Sichuan Medical College, Nanchong, China; 2 School of Management, North Sichuan Medical College, Nanchong, China; 3 School of Foreign Language, North Sichuan Medical College, Nanchong, China; 4 School of Public Health, North Sichuan Medical College, Nanchong, China

## Abstract

**Background:**

The prevalence of cognitive impairment among middle-aged and older adults remains high. While it has been proven that cigarette smoke exposure is associated with cognitive impairment, limited research has examined its relationship with the cognitive function trajectories of middle-aged and older adults.

**Methods:**

We included data on 5084 middle-aged and older adults from the China Health and Retirement Longitudinal Study (CHARLS), version D, which covers the latest surveys from 2011 to 2018. In the CHARLS, cognitive function was measured by the Chinese version of the Mini-Mental State Examination (MMSE). Individuals exposed to cigarette smoke were categorised into four levels: non-smokers, second-hand smokers, former smokers, and current smokers. We used the latent growth mixture model (LGMM) to identify the potential heterogeneity of cognitive trajectories, and an unordered multilevel logistic regression to explore the relationship between baseline cigarette smoke exposure and cognitive function trajectories.

**Results:**

We identified three cognitive trajectory groups: slow decline group (6.2%), stable group (84.6%), and rapid decline group (9.1%). After controlling for other variables, we found that current smokers were 1.429 times more likely to develop into the rapid decline group than non-smokers (odds ratio (OR) = 1.429; 95% confidence interval (CI) = 1.086–1.881). As we continued to include demographic factors as covariates, currents smokers were 1.454 times more likely to develop into the rapid decline group than non-smokers (OR = 1.454; 95% CI = 1.052–2.01). After we included social activities, drinking and health factors as covariates, current smokers were 1.414 times more likely to develop into the rapid decline group than non-smokers (OR = 1.414; 95% CI = 1.015–1.97). This meant that current smoking remained an independent risk factor for decline trajectories, even after accounting for demographics, social activities, and health factors, suggesting that smoking has a robust association with functional or health decline.

**Conclusions:**

The developmental trajectories of cognitive function among middle-aged and older adults are heterogeneous. We found that not smoking was a protective factor for cognitive function. This warrants further attention to the risk of cigarette smoking, which is a modifiable risk factor, and the subsequent adoption of interventions for smokers in order to slow down cognitive impairment and reduce its social and economic burden in the future.

The population of China is ageing rapidly, and this trend is expected to continue. Estimates indicated that, by 2050, there will be between 337 and 400 million older persons (≥65 years) in China [1]. Along with the ageing population, cognitive impairment has been recognised as a significant issue affecting the health of the country’s population. For example, it has been reported that people with mild cognitive impairment (MCI) have a 10-fold increased risk of developing Alzheimer’s disease (AD) compared with the general population [2]. Cognitive impairment is closely associated with dementia, which is the fifth leading cause of death globally. Importantly, the number of patients with dementia in China is projected to increase to 30 million by 2050 [3], which makes identifying potential risk factors and high-risk groups for cognitive impairment extremely important [4].

Previous studies have shown that the developmental trajectories of cognitive function among middle and old-aged adults are heterogeneous and influenced by various factors. Mose and colleagues [5] identified three cognitive function trajectory groups among Chinese adults aged ≥60 years: low-decline group (24.1%), medium-decline group (44.2%), and high-stable group (31.7%). They also found that social integration was significantly associated with the trajectories of cognitive function [5]. Zhang and colleagues [6] similarly identified three trajectories: slow decline (37.92%), rapid decline (6.71%), and stable function (55.37%). In their study, being overweight was related to the fast and slow decline trajectories, while obesity was associated with the slow decline trajectory of cognitive function [6]. Xie and colleagues [7] explored the association between childhood friendship status and cognitive ageing trajectory and found a significant relationship between the two in later life. Ding and colleagues [8] found that hypertension diagnosed in mid-life was linked to worse cognition. Xie and colleagues [9] examined the association between internal migration and cognitive function trajectories. They found that all cognitive function scores declined over time, but observed no significant differences in rates of cognitive decline between migrants and non-migrants [9].

However, less attention has been paid to the relationship between smoke exposure status and cognitive function trajectories among middle and old-aged adults. Considering this, we aimed to investigate the relationship between smoke exposure and cognitive trajectory and attempted to provide effective prevention methods for high-risk groups with cognitive impairment.

The prevalence of smoking in China is very high, with 350 million active smokers and 740 million secondhand smokers in 2010 [10]. Research has found that smoking not only directly damages the smoker's health, but also increases the incidence of different diseases among nonsmokers through passive or second-hand smoking [10,11]. However, some studies have found that smoking may not necessarily be exclusively negative. Nicotine has been shown to improve hippocampus-dependent learning in acute smoking [12] and to enhance attention, verbal memory, working memory, and executive function [13]. Despite these, numerous studies have demonstrated that active smoking has neurotoxic effects and significantly increases the risk of cognitive impairment [9,14,15] and dementia [16,17] among older people, and that it is associated with approximately a doubling in dementia risk for older adults [18,19]. There is also evidence that exposure to second hand smoke is associated with an increased risk of cognitive impairment in older adults [10,17], leading to significant increases in the risk of Alzheimer’s disease and vascular dementia [20]. Recent research has also found that longer second hand smoke exposure is related to greater declines over time in memory scores [21].

However, most previous studies investigating the relationship between smoke exposure and cognitive function were cross-sectional, with few adopting longitudinal designs and focussing on older Chinese people. Here we used data from a large sample from four waves (2011–18) of the China Health and Retirement Longitudinal Study (CHARLS) and proposed a method based on the latent growth mixture model (LGMM) to identify cognitive trajectories. We then used unordered multinomial logistic regression to explore the association of smoke exposure with cognitive function trajectories among middle and old-aged adults. Additionally, we explored whether this association is modified by factors such as age, sex, education, body mass index (BMI), residence, social activities, drinking, chronic disease, and depression.

## METHODS

### Data and study sample

We retrieved data from the CHARLS dataset and codebook, developed and maintained by the National Institute on Aging in China. The survey was first launched in 2011 and followed up in 2013, 2015, and 2018. The harmonised version D of CHARLS incorporates the latest data and adds variables for the fourth wave of 2018, containing 25 586 observations. The CHARLS programme received ethical approval from the Peking University Institutional Review Board [22]. All participants in the CHARLS provided written informed consent.

**Figure 1:**
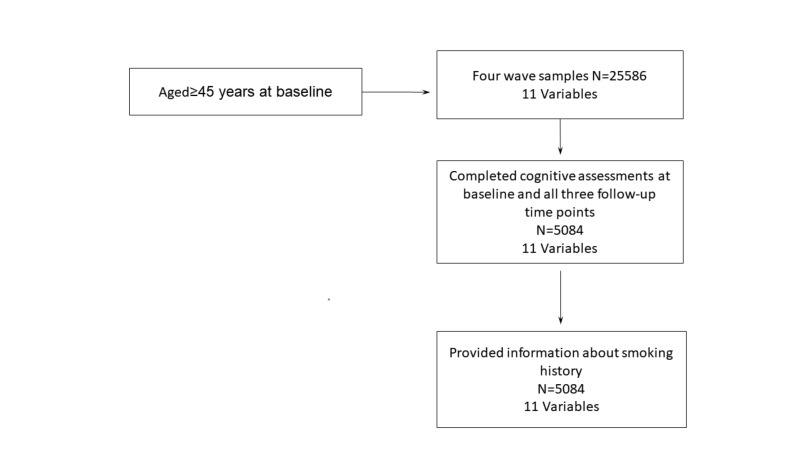
Flow diagram of sample selection.

In this study, middle-aged and older participants were from four waves of Harmonized CHARLS. We restricted our sample to 5084 respondents who met the following criteria: aged ≥45 years at baseline; completed cognitive assessments at baseline and all three follow-up time points; provided information about smoking history (**Figure 1**). We considered 11 different input variables in our analysis.

### Assessment of cognitive function

We obtained cognitive function data from baseline and all three follow-up assessments. According to previous studies derived from the CHARLS, cognitive function was measured by the Chinese version of the Mini-Mental State Examination (MMSE), which assesses two dimensions: episodic memory and mental status [9,23]. In short, the respondents heard a list of 10 unrelated Chinese words, were asked to repeat them immediately (immediate word recall), and again four minutes later (delayed word recall). The episodic memory score (range: 0–20 ) was calculated as the sum of the immediate recall and delayed recall scores, with a higher score indicating better memory.

Mental status was assessed using three dimensions: orientation, pentagon drawing test, and mathematical performance. Orientation measures included recalling the date (day, month, and year) and the day of the week, with scores ranging from zero to four. For the pentagon drawing test, participants were asked to redraw a picture of two overlapping pentagons; if the respondent failed to do so, the score was zero; if they drew it correctly, the score was one. In the serial subtraction, *i.e.* mathematical performance test, the respondents were asked to subtract seven from 100 and then continue subtracting seven from each answer for four more trials. Scores ranged from zero to five, with five indicating that all questions were answered correctly. The total score of all items ranged from 0 to 30 [24], with a higher score indicating better cognitive function.

### Assessment of smoke exposure

We divided our sample into four groups (0 = non-smokers, 1 = second hand smoker, 2 = former smokers, 3 = current smokers) based on answers to two questions from the CHARLS. One question was whether the respondent (*R1smokev*) or the spouse (*S1smokev*) had ever smoked. A code of 0 indicates that the respondent or the spouse reported never having smoked, while a code of 1 indicates that the respondent or the spouse reported smoking at some point. We categorised respondents and spouses scoring zero as non-smoking households and those scoring one into a group of smoking households. If a spouse in a smoker’s household did not smoke, they were considered a second-hand smoker, while the other was considered a smoker. We further divided smokers into former smokers and current smokers based on another question about whether they currently had a smoking habit (*R1smoken*, *S1smoken*). This question was only asked of respondents who reported ever smoking. A code of 0 indicates that the respondent had quit smoking, and a code of 1 indicates that the respondent was still smoking. In CHARLS, the smoking exposure was limited to the respondents and spouses, regardless of other members of the family. The formula is as follows:

Equation set 1:

*if R1smokev* + *S1smokev* = *0 and H1smokev* = *0;R1*(*Non-smoker*)

*if R1smokev* + *S1smokev* = *1 and H1smokev* = *1*

*if H1smokev* = *1 and R1smokev* = *1;R1smokev*(*Smoking*)*;S1smokev*(*Second-hand smoker*)

*if H1smokev* = *1 and R1smokev* = *0;R1smokev*(*Second-hand smoker*)*;S1smokev*(*Smoking*)

*if H1smokev* = *2;R1smokev*(*Smoking*)*;S1smokev*(*Smoking*)

Equation set 2:

*if R1smokev* = *1;H1smokev* = *1 OR 2;and Now:R1smoken* = *0*

*R1*(*Former smoker*)

*if R1smokev* = *1;H1smokev* = *1 OR 2;and Now:R1smoken* = *1*

*R1*(*Current smoker*)

### Assessment of covariates

We included the following variables as covariates in this research: age (1 ≤ 54, years, 2 = 55–64 years, 3 ≥ 65 years), gender (1 = male, 2 = female), educational level (1 = elementary school and below, 2 = middle school and above), residence (0 = urban, 1 = rural), chronic diseases (0 = none, 1 = one or two diseases, 2 = three or more diseases), social activities (0 = no, 1 = yes), drink (0 = no, 1 = yes), BMI, and the Center for Epidemiologic Studies Depression Scale (range 0–30). Respondents with a score of 10 or higher were likely to be suffering from significant depressive symptoms [25].

### Statistical analysis

We descriptively summarised individual demographics and related variables at baseline using means and standard deviations (SDs) for continuous and percentages for categorical variables. We used the χ^2^ test or analysis of variance F-test to compare the data between different trajectory groups.

A latent growth mixture model (LGMM) identified trajectories of MMSE scores, which provided two factors – intercept and slope – for describing the developmental trajectory characteristics, measured by mean and variance. The mean of the intercept factor represents the mean initial state of the corresponding category group, while its variance describes the degree of dispersion between individuals within the category at a specific time. The mean of the slope factor describes the mean rate of change of the category at different measured time points, while its variance represents the difference in the rate of change within the category.

We used the LGMM to analyse the longitudinal data and explore the heterogeneity. Through this method, we identified several potential subgroups of middle and older-aged adults, each characterised by unique developmental trajectories. In model selection, we considered many factors to ensure the best fit for our data. These factors included key statistical measures such as the *P*-values of model parameters and the confidence intervals (CIs) of trajectory estimates. Additionally, we evaluated the model fit using the Akaike information criterion (AIC) and Bayesian information criterion (BIC), where lower values indicated a better fit. The entropy index was used to assess classification accuracy. Furthermore, the likelihood ratio test Lo-Mendell-Rubin and the Bootstrap-based likelihood ratio test values reached significant levels (*P* < 0.05), indicating that model K explained more variance than model K-1 and that it fits better. We conducted these analyses in Mplus, version 8.3 (Muthén & Muthén, Los Angeles, California, USA).

We used unordered multinomial logistic regression to explore the relationship of baseline smoke exposure with cognitive function trajectories and the influences of covariates on the association between smoke exposure and cognitive function trajectories.

We tested three models: model 1 was not adjusted for any factors, model 2 was adjusted for age, sex, education level, and residence, and model 3 built upon model 2 by further adjusting for drinking, social activities, chronic disease, and depression. We performed these analyses using SPSS, version 27.0 (IBM Corp., Armonk, New York, USA). A *P*-value ≤0.05 indicated statistical significance.

## RESULTS

### Descriptive statistics

The baseline survey included 5084 respondents recruited in wave 2011. Among them, 1197 (26%) reported as non-smokers, 1455 (31.5%) as second-hand smokers, 439 (9.5%) as former smokers, and 1521 (33%) as current smokers at baseline. Further, 53.9% were men and 46.1% were women. In terms of education, 50.3% of the sample had middle school education or above. For age, 46.9% of respondents were aged 45–54 years, while in terms of residence, more than two-thirds of them lived in rural areas. Approximately 46.1% reported having fewer than three diseases, 42.2% reported participating in social activities, and 51.5% reported drinking alcohol. The mean BMI of the sample was 24.52. The mean score for depression in the slow decline group was 10.17, exceeding the critical score of 10. The mean MMSE score was 16.48 out of 30 (SD = 4.05) at baseline (2011); cognitive function increased in 2013, with a mean of 16.66, but decreased to 16.18 in 2015 and to 16.12 in 2018, with both values being lower than the baseline in 2011 (**Table 1**).

**Table 1:** Baseline characteristics of participants by different trajectory groups

	Total sample (n = 5084)	Stable group (n = 4302, 84.6%)	Slow decline group (n = 317, 6.2%)	Rapid decline group (n = 465, 9.1%)	Test of association (χ^2^)	*P*-value
**Smoking**					13.33	0.038
Non-smoker	1197 (26)	1052 (26.7)	57 (21.8)	88 (21.5)		
Second-hand smoking	1455 (31.5)	1237 (31.4)	96 (36.8)	122 (29.8)		
Former smoker	439 (9.5)	373 (9.5)	20 (7.7)	46 (11.2)		
Current smoker	1521 (33)	1280 (32.5)	88 (33.7)	153 (37.4)		
Missing value	472	360	56	56		
**Gender**					10.46	0.005
Male	2739 (53.9)	2344 (54.5)	143 (45.1)	252 (54.2)		
Female	2345 (46.1)	1958 (45.5)	174 (54.9)	213 (45.8)		
**Age in years**					222.64	<0.001
45-54	2378 (46.9)	2174 (50.6)	93 (29.5)	111 (23.9)		
55-64	1944 (38.3)	1594 (37.1)	139 (44.1)	211 (45.4)		
≥65	753 (14.8)	527 (12.3)	83 (26.3)	143 (30.7)		
Missing value	9	7	2	0		
**Education level**					419.51	<0.001
Elementary school and below	2526 (49.7)	1878 (43.7)	287 (90.5)	361 (77.6)		
Middle school and above	2558 (50.3)	2424 (56.3)	30 (9.5)	104 (22.4)		
**Rural/urban**					77.44	<0.001
Urban	1336 (26.3)	1228 (28.6)	33 (10.4)	75 (16.1)		
Rural	3746 (73.7)	3072 (71.4)	284 (89.6)	390 (83.9)		
Missing value	2	2	0	0		
**Social activities**					25.632	<0.001
No	2465 (48.5)	2030 (47.2)	194 (61.2)	241 (51.9)		
Yes	2618 (51.5)	2272 (52.8)	123 (38.8)	223 (48.1)		
Missing value	1	0	0	1		
**Drink**					0.458	0.795
No	2939 (57.8)	2493 (58)	184 (58)	262 (56.3)		
Yes	2144 (42.2)	1808 (42)	133 (42)	203 (43.7)		
Missing value	1	1	0	0		
**Chronic disease**					4.95	0.293
None	2093 (41.2)	1796 (43.1)	126 (41.4)	171 (38.1)		
One-two diseases	2343 (46.1)	1966 (47.1)	144 (47.4)	233 (51.9)		
Three or more diseases	487 (9.6)	408 (9.8)	34 (11.2)	45 (10)		
Missing case	161	132	13	16		
**BMI, mean (SD)**	24.52 (36.48)	24.77 (39.81)	23.25 (3.84)	23.27 (3.73)	0.514	0.598
**CESD, mean (SD)**	7.24 (5.81)	6.95 (5.65)	10.17 (6.93)	8 (5.88)	50.75†	<0.001
**MMSE score in 2011, mean (SD)**	16.48 (4.05)	16.97 (3.77)	10.19 (2.49)	16.26 (3.52)	495.75†	<0.001
**MMSE score in 2013, mean (SD)**	16.66 (4.06)	17.29 (3.69)	9.87 (2.97)	15.47 (3.42)	645.32†	<0.001
**MMSE score in 2015, mean (SD)**	16.18 (4.07)	17.61 (3.47)	9.61 (2.97)	12.07 (3.12)	1086.82†	<0.001
**MMSE score in 2018, mean (SD)**	16.12 (4.95)	17.51 (3.86)	8.76 (2.90)	8.22 (2.77)	1956.90†	<0.001

BMI – body mass index, CESD – Center for Epidemiologic Studies Depression Scale, MMSE – Mini-Mental State Examination, SD – standard deviation

*Values are presented as n (%) unless specified otherwise.

†F-value of independent F-test.

### Fitting information LGMM

We extracted cognitive function trajectories of 1 to 4 potential categories from the initial model, respectively. The values of the Akaike information criterion and the Bayesian information criterion became smaller as the number of classifications increased in the first three categories. Both the Lo-Mendell-Rubin and Bootstrap-based likelihood ratio values were significant (*P* < 0.01) (**Table 2**). Considering the numerical indicators of entropy and category probability, we selected the classification of three latent categories as the optimal category model. The average probability of the subject belonging to each potential category in each category was between 60.7% and 88.2%, indicating that the results of the three potential categories are suitable (**Table 3**).

**Table 2:** LGMM model fitting information for different categories

Category number	K	Kog(L)	AIC	BIC	Entropy	LMR	BLRT	Conditional probability
1	12	−55434.95						
2	15	−55377.02	110784.04	110882.05	0.569	0.0000	0.0000	0.22/0.78
3	18	−55362.77	110761.53	110879.14	0.625	0.0003	0.0004	0.06/0.85/0.09
4	21	−55352.39	110764.78	110883.99	0.703	0.0000	0.0000	0.84/0.001/0.06/0.1

AIC – Akaike information criterion, BIC – Bayesian information criterion, BLRT – bootstrap-based likelihood ratio test, LMR – Lo-Mendell-Rubin likelihood ratio test

**Table 3:** Average attribution probability (column) of each potential category sample (row)

Potential category groups	Slow decline group, %	Stable group, %	Rapid decline group, %
Slow decline group	60.7	19.3	20
Stable group	5.2	88.2	6.7
Rapid decline group	17.9	19.1	63

### Cognitive development trajectory for each latent category

The fitted information index of the LGMM for each latent category showed that the three-category model is optimal, and that the developmental trajectory can be distinguished into three different latent categories. In the slow decline group, the mean initial level of cognitive function at the first test was 12.456, while the slope factor indicated that it decreased over the four tests at a rate of 1.236. In the stable group, the mean initial level of cognitive function at the first test was 17.132; the slope factor indicated that it remained stable over the four tests. In the rapid decline group, the mean initial level of cognitive function at the first test was 16.224, while the slope factor indicated it declined rapidly over the four tests, with a rate of 4.321 (Table S1 in the **Online Supplementary Document****)**.

**Figure 2:**
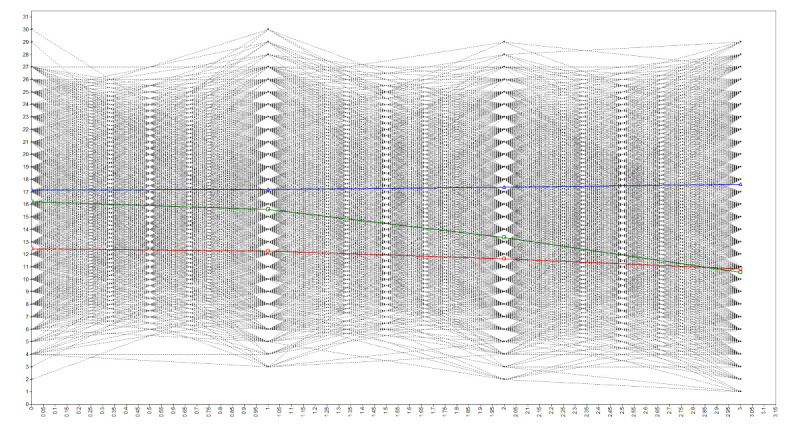
Estimated mean and trajectory of each potential category.

According to the statistical results of the mean values of the intercept factor and slope factor, the initial level and slope were all quite different (**Figure 2**). We thus named each category's initial level and rate of change descriptively. Specifically, the stable and rapid decline groups showed relatively high initial levels, but their change trends differed. The cognitive function of the stable group showed a stable trend, while the rapid decline group showed a rapid downward trend. The cognitive function in the slow decline group showed a relatively low initial level, with a slow downward change trajectory. We therefore named the three potential categories of cognitive function as follows:

1. Slow decline group: a slow decline trend of the initial cognitive level over the four measurement time points.
2. Stable group: initial stability of cognitive function over the four measurement time points.
3. Rapid decline group: rapid decline trend of a high initial cognitive level over the four measurement time points.

The variance of the intercept factor was 5.51 (*P* = 0.000), indicating that the initial values of cognitive function differed within each category. However, the variance of the slope factor was −0.798 (*P* = 0.111), indicating that the difference in cognitive decline rate among individuals was not significant within each category.

### Influences of covariates on the association between smoke exposure and cognitive function trajectories

We used an unordered multinomial logistic regression to explore the relationship between baseline smoke exposure and cognitive function trajectories (Table S2 in the **Online Supplementary Document**).

First, compared to the stable group, after controlling for other variables, we found that current smokers were 1.429 times more likely to develop into the rapid decline group than non-smokers (odds ratio (OR) = 1.429; 95% confidence interval (CI) = 1.086–1.881). Meanwhile, second-hand smokers were 1.432 times more likely to develop into the slow decline group than non-smokers (OR = 1.432; 95% CI = 1.022–2.008). Former smokers were 1.474 times more likely to develop into the rapid decline group than non-smokers (OR = 1.474; 95% CI = 1.013–2.146).

After adjusting for some demographic factors (age, gender, education level, residence, BMI), we found that current smokers were 1.531 times more likely to develop into the slow decline group than non-smokers (OR = 1.531; 95% CI = 1.014–2.313), and 1.454 times more likely to develop into the rapid decline group than non-smokers (OR = 1.454; 95% CI = 1.052–2.01).

Finally, after further adjusting for lifestyle, and health factors (social activities, drinking, and chronic disease covariates), we found that current smokers were 1.414 times more likely to develop into the rapid decline group than non-smokers (OR = 1.414; 95% CI = 1.015–1.97).

## DISCUSSION

In this national longitudinal study in China, we explored the association between smoke statuses and cognitive trajectories of a nationally representative sample of 5084 Chinese middle and old-aged adults (age ≥45 years). We found three different trajectories of cognitive function, categorised as the stable, slow decline, and rapid decline groups. We further observed that not smoking is a protective factor for cognitive function, and that middle and old-aged adults non-smokers are more likely to have a higher stable trajectory of cognitive function than those who currently smoke.

The study showed that middle- and older adults have low cognitive function scores and a downward trend with age. This finding is consistent with previous research [26]. Singh-Manoux and colleagues [27] found that cognitive decline was already evident in the middle-aged population across five age categories (45–49, 50–54, 55–59, 60–64, and 65–70 at baseline), showing faster decline in older people. This might be due to several reasons. First, age-related hearing loss is a common problem for older adults, leading to cognitive decline because cortical resources are strained, potentially changing how the brain responds to cognitively demanding situations [28]. Second, the proportion of hypertension in the elderly increases with age. Some evidence has shown that hypertension is a significant risk factor for cognitive impairment in middle and older adults [29]. Increased blood pressure variability has been related to an increased risk of dementia [30]. Third, the functional capabilities of the brain decline progressively during aging, generally resulting in decreased cognitive performance [31].

Here we found that most respondents belonged to the stable trajectory group (84.6% of the sample), compared to the slow decline group (6.2% of the sample) and the rapid decline group (9.1% of the sample). These results are consistent with prior studies [6]. Compared with the stable group, we found that not smoking was a protective factor for both the slow decline group and the rapid decline group, while current smoking was a risk factor. The results align with prior studies. For example, Amin and colleagues [32] found that smoking can have a long-term impact on both executive function and memory among older adults; specifically, current smokers had an increased risk of executive function impairment compared to former smokers, and lung diseases and smoking can synergistically influence executive function. Anstey and colleagues also found that smoking may be a risk factor for cognitive decline and dementia [16]. In our sample, current smokers showed a significantly larger yearly decline in MMSE scores compared with non-smokers over the follow-up period.

Several reasons might explain these findings. For example, an inflammatory response is a potential mechanism explaining the relationship between smoking and cognitive function, potentially leading to adverse effects on cognitive function [33]. Meng and colleagues [34] found that smoking exacerbated cognitive impairment in a rat model of vascular dementia through neuroinflammation. Some studies have also shown that current smokers have decreased grey matter density in areas critical to cognitive function [18] and exhibited cortical thinning in frontal and temporoparietal regions [35] compared with never-smokers. Simultaneously, smoking can increase the risk of chronic diseases, which may lead to cognitive changes. For example, some studies found smoking increased the risk of cardiovascular disease [36], chronic obstructive pulmonary disease [37], diabetes [38], and other chronic diseases [39]. Loretan and colleagues [40] found that smoking may persist among adults with chronic diseases and is most prevalent among adults with two or more chronic diseases. Other research showed that chronic diseases may cause alterations in brain structure and function and are related to cognitive changes [41], with some changes potentially associated with neurodegenerative diseases.

We found that factors such as being older, being less educated, having less social activities, residing in rural areas, and having depressive symptoms were associated with a decline in cognitive function [42,43] and modified the relationship between smoking status and cognitive function. The Lancet Commission on Dementia Prevention, Intervention, and Care proposed a life course model of dementia risk, suggesting that approximately 40% of dementia worldwide is attributable to 12 modifiable risk factors at different phases of life [44]. Three of these factors are low education, depression, and smoking. Older adults receiving higher education may be involved in more cognitive activities to improve their cognitive reserve, enhancing the brain's coping ability or compensating for neuropathological damage. Therefore, education can improve people's cognitive reserve and buffer against cognitive decline [45]. However, in this study, it was also found that the educational level of respondents in rural areas was relatively lower compared with urban respondents.

Similarly, compared with urban residents, the respondents in our sample who resided in rural areas were less likely to participate in social activities. We thus found that individuals with limited social engagement and lower educational attainment were more likely to experience cognitive decline. Aside from this, we should note that depression in old age is a risk factor for dementia and may also be a prodromal symptom or early stage of dementia. Studies have found that depression affects glucocorticoids, neuronal growth factors, and hippocampal volume, which in turn leads to cognitive dysfunction. Smoking increases cardiovascular pathological damage and neurotoxic substances, which can damage tissue neurons and lead to cognitive dysfunction [34]. In many regions of China, especially rural communities, offering cigarettes to guests is a common and highly-valued social custom [47]. The spouses of smokers are more likely to be passive smokers at home because their husbands or other family members smoke cigarettes [21]. Therefore, to prevent an increasing prevalence of cognitive decline, we suggest that health authorities should pay more attention to the risk of smoking, which is a modifiable risk factor. Furthermore, medical institutions should also focus on older adults, those who engage in fewer social activities, those who are less educated, and rural groups.

A significant strength of this study is our use of a large representative sample and longer-term panel data to test the connections between smoking exposure and cognitive trajectory, thus increasing the study's external validity. Additionally, we used the LGMM to analyse cognitive trajectories, compensating for traditional growth models' deficiency in exploring cognitive heterogeneity among middle-aged and older adults. This model assumes multiple potential cognitive growth trajectories exist in the population, with each potential trajectory representing a subclass with different growth patterns for different subclasses. This allowed us to explore trends in smoke status and cognitive function decline with age at multiple time points.

This study also has a few limitations. First, although we adjusted for a large number of confounders as much as possible, there were also potential residual confounders, including unmeasured variables excluded due to statistical constraints (*e.g.* medication usage) and unavailable data in the original database (*e.g.* air pollution exposure). These omitted factors could have introduced bias into our findings. Second, our primary analysis included only those who completed the baseline and all three follow-up surveys to measure cognitive trajectories, which might have led us to ignore the follow-up of participants with different characteristics. This is highly likely to have trigger a selection bias. Simultaneously, our reduced sample size reduced our statistical power. Third, this study has a limitation due to our approach to coding the second-hand smoking exposure: household second-hand smoke exposure was inferred solely based on the smoking status of respondents and their spouses, without accounting for smoking behaviours of other household members such as children and relatives. This simplified approach may have resulted in exposure misclassification. Fourth, we did not include data on key details such as the number of cigarettes smoked per day and smoking duration, which may have led to ambiguity of exposure heterogeneity. At the same time, it also showed that the quality of our data needs to be improved, which is a could be considered in future studies.

## CONCLUSIONS

We found that the developmental trajectories of cognitive function among middle and old-aged adults are heterogeneous, with most respondents belonging to the stable trajectory group. Compared with the stable group, we found that smoking may be a marker of cognitive decline, and not smoking was a protective factor against cognitive decline. When adjusting for age, sex, education, place of residence, chronic disease, social activities, BMI, drink, and depressive symptoms as covariates, we found that not smoking remained a protective factor against cognitive decline, while current smoking was a risk factor for both the slow-decline group and the rapid-decline group.

These findings highlight the need to pay more attention to the risk of current smoking, which is a modifiable risk factor. Furthermore, we should focus more on the elderly, those with less social activities, the less educated, and rural groups, and adopt targeted interventions for these populations. Therefore, in terms of health education, rural broadcasts and bulletin boards could be used to promote the health hazards of smoking. Individuals with less social activity could be additionally included into social activities through the organisation of community health lectures and smoking cessation support group activities, which would also help with disseminating the knowledge about the harms and benefits of smoking cessation. Regarding environmental improvement, rural public places should be encouraged to set up clearly visible non-smoking signs. At the same time, to reduce second-hand smoke exposure at the family, we should provide professional smoking cessation counselling, promote the creation of smoke free families, and launch family health promotion programmes. From the perspective of resource support, considering the relatively weak medical resources in rural areas, medical teams can be organised to go to the countryside to provide regular smoking cessation consultation and guidance services.

Our findings also delineate three critical directions for future investigations. First, studies should be expanded to integrate additional confounding factors systematically. Second, direct measurement of individual-level second-hand smoking exposure biomarkers is imperative to refine exposure assessment precision. Finally, comparative regional analyses could be incorporated to examine geographic heterogeneity in smoking-related cognitive outcomes.

## Additional material


Online Supplementary Document
