# Supplementary material for: Association of smoke exposure with cognitive function trajectories among middle and old-aged adults: evidence from the China Health and Retirement Longitudinal Study
Source: J Glob Health. 2025 May 5;15:04150. doi: 10.7189/jogh.15.04150 (PMC12050903; doi:10.7189/jogh.15.04150)
Supplement: Online Supplementary Document [file jogh-15-04150-s001.pdf]

Supplement to: Rulin Li, Lanjun Luo, Changwan Yuan, Qi Zhu. Association of SmokeExposure with Cognitive Function Trajectories Among Middle and Old-AgedAdults: Evidence from the China Health and Retirement Longitudinal Study. JGlob Health. 2025;15:04150.

Table S1. Mean and variance of intercept (I) and slope (S) factors for each potential category

category	factor	B	SE	P
	Mean			
Slow decline group	I	12.456	0.527	0.000
	S	-1.236	0.423	0.003
	Mean			
stable group	I	17.132	0.097	0.000
	S	0.359	0.117	0.002
	Mean			
rapid decline group	I	16.224	0.482	0.000
	S	-4.321	0.385	0.000

SE -Standard Error

*B-B value of Beta Coefficient

Table S2. Association of smoke exposures with trajectories of MMSE scores

SLOW DECLINE VS	STABLE			RAPID	DECLINE VS	STABLE
Adjustment	OR	95%CI	P	OR	95%CI	P
**Model1**						
Smoking (Ref.non-smoker)						
current smoker	1.269	0.9, 1.788	0.174	1.429	1.086, 1.881	**0.011**
secondhand	1.432	1.022, 2.008	**0.037**	1.179	0.886, 1.57	0.259
former smoker	0.990	0.587, 1.669	0.969	1.474	1.013, 2.146	**0.043**
**Model 2**						
Smoking (Ref.non-smoker)						
current smoker	1.531	1.014, 2.313	**0.043**	1.454	1.052, 2.01	**0.023**
secondhand	1.149	0.767, 1.721	0.5	1.095	0.77, 1.558	0.614
former smoker	1.034	0.573, 1.866	0.912	1.447	0.955, 2.193	0.081
Gender (Ref. Female)						
Male	0.66	0.434, 1.002	0.051	0.822	0.582, 1.16	0.265
Age(Ref.≥65)						
45-54	0.352	0.237, 0.523	<0.001	0.218	0.158, 0.3	<0.001

55-64	0.512	0.358, 0.733	<0.001	0.440	0.333, 0.58	<0.001
BMI	0.975	0.938, 1.013	0.195	0.974	0.944, 1.004	0.092
Educational level (Ref. Middle school and above)						
Elementary school and below	8.986	5.688, 14.198	<0.001	2.929	2.246, 3.819	<0.001
residence(Ref.rural)						
urban	0.384	0.234, 0.632	<0.001	0.527	0.380, 0.730	<0.001
**Model 3**						
Smoking (Ref.non-smoker)						
current smoker	1.42	0.932, 2.164	0.103	1.414	1.015, 1.970	**0.04**
secondhand	1.089	0.716, 1.656	0.69	1.076	0.752, 1.539	0.688
former smoker	0.972	0.533, 1.775	0.927	1.344	0.873, 2.067	0.179
Gender (Ref. Female)						
Male	0.71	0.449, 1.122	0.142	0.794	0.546, 1.154	0.226
Age(Ref.≥65)						
45-54	0.335	0.221, 0.509	<0.001	0.213	0.153, 0.298	<0.001
55-64	0.507	0.350, 0.736	<0.001	0.436	0.328, 0.578	<0.001
BM1	0.989	0.949, 1.031	0.6	0.975	0.943, 1.007	0.125
Educational level (Ref. Middle school and above)						
Elementary school and below	9.084	5.63, 14.658	<0.001	2.866	2.189, 3.752	<0.001
residence(Ref.rural)						
urban	0.431	0.258, 0.722	0.001	0.551	0.396, 0.767	<0.001
Chronic disease(Ref. Three or more diseases)						
None	1.366	0.805, 2.317	0.247	1.158	0.753, 1.782	0.503
One-two diseases	1.052	0.634, 1.745	0.844	1.144	0.762, 1.716	0.517
Drink (Ref. Yes)						
No drink	0.869	0.620, 1.217	0.413	0.93	0.710, 1.218	0.598
Social activities(Ref. Yes)						
No social activities	1.500	1.124, 2.003	**0.006**	0.98	0.781, 1.230	0.863
CESD	1.062	1.038, 1.086	<0.001	1.017	0.997, 1.037	0.098

OR-odds ratio

CI -confidence interval

* model 1 was no adjustment.

†model 2 was based on model 1 plus baseline adjust for age, gender, educational level, residence and BMI.

‡model 3 was based on model 2 plus baseline social activities, drink, chronic diseases, and cesd-10.

Correspondence to:
Lanjun Luo
School of Management,
North Sichuan Medical College
Dongshun Road, Nanchong China
lanjun@nsmc. edu. cn
